# The relationship between oral health-related quality of life, the need for orthodontic treatment and bullying, among Brazilian teenagers

**DOI:** 10.1590/2177-6709.24.2.073-080.oar

**Published:** 2019

**Authors:** Renata Colturato Joaquim Gatto, Artênio José Ísper Garbin, José Eduardo Corrente, Cléa Adas Saliba Garbin

**Affiliations:** 1 Universidade Estadual Paulista, Programa de Pós-graduação em Odontologia Preventiva e Social (Araçatuba/SP, Brazil).; 2 Universidade Estadual Paulista, Instituto de Biociências, Departamento de Bioestatística (Botucatu/SP, Brazil).

**Keywords:** Adolescent, Quality of life, Bullying, Orthodontics, Malocclusion

## Abstract

**Introduction:**

Orthodontic treatment aims at oral health and restoration of function as main objectives, however, psychological and social effects end up being the main reason for the demand for treatment.

**Objective:**

To determine the association between the oral health-related quality of life (OHRQoL), the need for orthodontic treatment and bullying among Brazilian teenagers.

**Methods:**

This was a cross-sectional epidemiological study. To assess the malocclusion, the Dental Aesthetic Index was used. And the Oral Health Impact Profile-14 was used to analyze the OHRQoL. The Kidscape questionnaire was used to investigate cases of bullying. The following variables were also included: previous orthodontic treatment and a desire to fix the teeth to improve one’s appearance. Multivariate analysis was performed using logistic regression considering the poor OHRQoL as a response variable.

**Results:**

815 teenagers participated in the study. There was a statistically significant association between oral health-related quality of life and the variables: previous orthodontic treatment (*p*= 0.0270), desire to fix the teeth (*p*< 0.0001), sex (*p*= 0.0309), history of being a victim of bullying (*p*< 0.0001), frequency of bullying episodes (*p*= 0.0170), and consequences of bullying (*p*< 0.0001). The following were considered as risk factors for poor OHRQoL: lack of previous orthodontic treatment (OR = 2.191) and negative consequences of bullying (OR = 3.042).

**Conclusion:**

The need for orthodontic treatment was not associated with the OHRQoL; however, bullying and previous orthodontic treatment had a statistically significant association with this variable.

## RESUMO

**Introdução:**

o tratamento ortodôntico visa à saúde bucal e ao reestabelecimento da função como objetivo final; no entanto, os efeitos psicológicos e sociais acabam sendo o principal motivo da procura pelo tratamento.

**Objetivo:**

determinar a associação entre a qualidade de vida relacionada à saúde bucal (QVRSB), a necessidade de tratamento ortodôntico e o bullying entre os adolescentes.

**Métodos:**

trata-se de um estudo epidemiológico transversal. Para avaliar a má oclusão, foi utilizado o *Dental Aesthetic Index*. O instrumento *Oral Health Impact Profile-14* foi utilizado para analisar a QVRSB. O questionário *Kidscape* foi utilizado para verificar os casos de bullying. Também foram incluídas as variáveis: tratamento ortodôntico prévio e desejo de corrigir os dentes para melhorar a aparência. A análise multivariada foi realizada por meio de regressão logística, considerando-se a qualidade de vida ruim como variável resposta.

**Resultados:**

815 adolescentes participaram da pesquisa. Houve associação, estatisticamente significativa, entre a qualidade de vida relacionada à saúde bucal e as variáveis: tratamento ortodôntico prévio (*p*= 0,0270), desejo de corrigir os dentes (*p*< 0,0001), sexo (*p*= 0,0309), bullying (*p*< 0,0001), frequência de episódios de *bullying* (*p*= 0,0170) e consequências do bullying (*p*< 0,0001). Foram considerados como fatores de risco para uma QVRSB ruim os adolescentes que não realizaram tratamento ortodôntico prévio a esse estudo (OR= 2,191) e aqueles que revelaram consequências negativas relacionadas aos episódios de bullying (OR= 3,042).

**Conclusão:**

a necessidade de tratamento ortodôntico não foi associada à qualidade de vida relacionada à saúde bucal, já o bullying e o tratamento ortodôntico prévio tiveram associação estatisticamente significativa com essa variável.

## INTRODUCTION

Malocclusion is considered a public health problem, due to its high prevalence in the society.^1^ ^,^ ^2^ In Brazil, it ranks third among the major problems related to oral health, and about 37.6% of adolescents aged 12 years or older have some type of malocclusion^3^. Although it is not considered a disease, the disorder requires orthodontic treatment because it may increase the subject’s susceptibility to develop diseases such as periodontitis and trauma, and also affect oral functions, making it difficult to chew, swallow, and speak.^4^ ^,^ ^5^

Scientific evidence has shown that malocclusion can have a negative effect on the oral health-related quality of life (OHRQoL). Untreated people who have this disorder, in comparison to those who have already received orthodontic treatment, usually have a worse OHRQoL,^6^ ^,^ ^7^ since it is a complex condition that involves biological and psychosocial factors.^8^

Although orthodontists traditionally consider oral health and function reestablishment as the main objectives of clinical intervention, psychological and social effects can be the main reasons leading patients to seek for treatment.^9^

In this context, it is known that the expression of body image has great importance during adolescence. Dissatisfaction with the body at this stage can be quite prevalent, because physical changes and the significant biopsychosocial transformations inherent to this period of life can be potentially negative when individuals are morbidly concerned about how they are seen by the others.^10^ ^,^ ^11^ In addition dissatisfaction with body image may appear as a factor associated with bullying.^12^ ^,^ ^13^

Bullying among adolescents at school has been widely studied. This phenomenon is a subtype of violence, and it is characterized by negative actions of one or more students in an unequal power relationship.^14^

However, there are few reports of studies that evaluated the impact of bullying on OHRQoL of Brazilian adolescents. Furthermore, most of the studies investigating the effect of orthodontic treatment on OHRQoL in adolescents were conducted in a clinical setting, by evaluating adolescents who seek treatment for either aesthetic or functional reasons. In this context, the objective of this study was to determine the association between the OHRQoL, the need for orthodontic treatment and a history of bullying, among Brazilian adolescents enrolled in the public education system. 

## MATERIAL AND METHODS

### Study design and sample selection

This was a cross-sectional epidemiological study with adolescents of both sexes enrolled in public schools of a medium-size city in northwest São Paulo, Brazil, from August to December 2014. The city had 20 elementary schools in the urban area, and nineteen schools were selected to be part of this study, because one of them did not consent in time for data collection (n = 4.283). The students participating in the study belonged to the 11-to-16-year-old age group (students from 7^th^, 8^th^, and 9^th^ grades of elementary school). This age group was chosen because they had the minimum education required to answer the self-administered questionnaire. All adolescents enrolled in these grades were invited to participate in the study; however, were excluded: adolescents whose parents did not allow the examinations; adolescents who were allowed but did not want to participate; and adolescents who were not present on the three dates scheduled for the examinations.

Considering the age range of the students, the prevalence of bullying in the city studied was unknown (50%). Considering an error margin of 5% and reliability of 95%, the minimum sample size was determined to be 384 individuals. Due to the number of schools in the city, the sample collection was performed considering both the number of schools and the number of students. It was established that the effect of the sampling design was two-fold (corresponding to the two collection stages), and, in this case, the sample size should be at least 768 individuals.

The final sample consisted of 815 adolescents; therefore, it showed adequate statistical power. Moreover, the bullying sample was estimated to be 48.22%, which is close to the prevalence established and within the error margin considered.

### Data collection

Initially, participants were separated into groups of 10 students and asked to answer to the self-administered questionnaire (assessing the variables sex, age, skin color, history of orthodontic treatment prior to the study, desire to fix the teeth to improve one’s appearance) and the Oral Health Impact Profile (OHIP-14) and Kidscape indices. The OHIP-14 index measures the impact of dental problems on the subject’s quality of life. Its first version was developed by Slade and Spencer ^15^ in 1994, and contained 49 questions (OHIP-49); later, Slade^16^ (1997) published a reduced form containing 14 questions (OHIP-14). 

Oliveira and Nadanovky^17^ (2005) performed the instrument validation in Portuguese, describing the psychometric properties of the Brazilian version of OHIP-14. The result of this index can range from 0 to 56. The minimum value obtained in this study was 0 and the highest, 32. It was considered the median as the cutoff point; thus, OHIP< 8 was considered as good and ≥8, as bad.

In order to evaluate bullying among students, we used the Kidscape model, a questionnaire developed by the British institution KIDSCAPE, which is engaged in preventing bullying and child sexual abuse.^18^ The questionnaire was adapted for this study, being considered the following issues: the frequency of bullying episodes, its consequences, the type of intimidation, the sex of the author, and if the victim ever practiced bullying. For the question regarding the sex of the author of the bullying, the following categories were added: boy, girl, no answer. For the question regarding the kind of bullying suffered, were included options to provide more than one answer and no answer. 

Subsequently, oral examinations were performed following the diagnostic criteria recommended by the World Health Organization (WHO). Biosecurity precautions were taken to protect the participants and the examiner. The Dental Aesthetic Index (DAI) was used, an instrument proposed by Cons et al^19^in 1989, which evaluates, besides the occlusion, the aesthetics of the individual. After examination, the occlusal condition is categorized into the following groups: no abnormality or mild malocclusion (without treatment need), defined malocclusion, severe malocclusion, and very severe or disabling malocclusion. To analyze the association of data, the results of this index were dichotomized in: need for treatment (> 25: mild, moderate and severe) and no need for treatment (≤ 25: normal).

The oral examination and the questionnaire analysis were carried out in the educational institution itself by a single previously calibrated researcher. The pilot study and the pre-test preceded the study.

### Data analysis

Data were analyzed using the statistical program SAS for Windows, version 9.3. The chi-square test or Fisher’s exact test at a 5% level of significance was applied to verify the association between OHRQoL and the independent variables (sex, age, skin color, DAI index, previous orthodontic treatment, desire to fix the teeth, history of being a victim of bullying, frequency of bullying episodes, sex of the author, the kind of bullying suffered, and if the victim has ever practiced bullying). Multivariate analysis was performed using logistic regression considering poor quality of life as a response variable, and the others as explanatory variables. Variables that showed statistical significance were included in the model and the results were expressed as an odds ratio (OR), in association with bad OHRQoL and its confidence interval of 95% (CI).

### Ethical aspects

The study was approved by the Ethics Committee in Research with Human Subjects of *Universidade Estadual Paulista Júlio de Mesquita Filho*. The adolescents were examined only when parents or guardians had signed the Informed Consent form. Data collection began after the approval of the regional director of education and the direction of the schools involved.

## RESULTS

A total of 815 adolescents aged between 11 and 16 years, and enrolled in municipal elementary schools participated in the study. Table 1 shows the epidemiological profile of the students; the age distribution shows that the extremes of 11 and 16 years were the least prevalent. The study population predominantly contained participants with brown skin and female participants. Table 1 also shows that most students had not undergone orthodontic treatment prior to this study, although almost all of them reported that they would like to fix the teeth to improve their appearance. Approximately 40% of the studied adolescents had a need for orthodontic intervention, and the relationship of those who considered having a good OHRQoL with those who did not consider was balanced.

**Table 1:** Absolute and percentage distribution of the variables age, sex, skin color, Dental Aesthetic Index and Oral Health Impact Profile in adolescents enrolled in public schools.

Variables	n	%
Age (years)		
11	14	1.7
12	254	31.2
13	225	27.6
14	259	31.8
15	55	6.7
16	8	1.0
Sex		
Female	488	59.9
Male	327	40.1
Skin color		
White	333	40.9
Black	97	11.9
Brown	385	47.2
Previous orthodontic treatment		
Yes	190	23.3
No	625	76.7
Would like to correct the teeth to improve the appearance?		
Yes	745	91.4
No	70	8.6
Dental Aesthetic Index (DAI)		
≤ 25 (no abnormality or mild malocclusion)	491	60.2
26-30 (defined malocclusion)	179	22.0
30-35 (severe malocclusion)	78	9.6
≥ 36 (severe or crippling malocclusion)	67	8.2
Oral health impact profile (OHIP- 14)		
Good (≤ 8)	439	53.9
Bad (> 9)	376	46.1

Table 2 shows an alarming fact: almost half (48.2%) of the adolescents interviewed reported being victims of bullying. In most cases, the event was not isolated and was repeated more than once. Notoriously, male adolescents were the main provocateurs of the episodes of bullying. According to the victims, the intimidation was usually in the form of verbal aggression. Students who experienced bullying, in most cases, did not declare themselves as authors of episodes of intimidation.

**Table 2:** Absolute and percentage distribution of variables related to episodes of bullying in adolescents enrolled in public schools.

Variables	n	%
Victim of bullying		
Yes	393	48.2
No	422	51.8
Frequency of suffering bullying		
One time	137	34.8
Several times	161	41.0
Almost every day	57	14.5
Several times a day	38	9.7
Consequences of bullying		
No consequences	214	54.3
Some bad consequences	140	35.5
Terrible consequences	22	5.9
Had to change school	17	4.3
Sex of antagonist		
Boy	256	65.1
Girl	91	23.2
Boys and girls	44	11.2
No response	2	0.5
Type of bullying suffered		
Emotional	36	9.2
Verbal	183	46.7
Physical	55	14.0
Racist	48	12.3
Sexual	11	2.8
More than one response	58	14.8
No response	2	0.2
Protagonist of bullying		
Yes	127	32.3
No	266	67.7

Source: Gatto et al.^20^

Table 3 shows the relationship between the variables Dental Aesthetic Index and the history of being a victim of bullying.

**Table 3:** Association between the Dental Aesthetic Index and victims of bullying variable.

Dental aesthetic index (DAI)	Victim of bullying, n (%)	Non-victim of bullying, n (%)	P value
26-30 (defined malocclusion)	81 (51.5)	98 (59.0)	0.3327
30-35 (severe malocclusion)	40 (25.2)	38 (22.9)	
≥ 36 (very severe or disabling malocclusion)	37 (23.3)	30 (18.1)	

Analysis of the association between OHRQoL and other independent variables (Table 4) shows a statistically significant association between OHRQoL and: sex, orthodontic treatment performed prior to data collection, desire to fix the teeth to improve one’s appearance, history of being a victim of bullying, frequency of episodes of bullying, and negative consequences related to bullying (*p*< 0.05). 

**Table 4:** Association of oral health-related quality of life with other dependent variables.

Variables	Good OHRQoL, n (%)	Bad OHRQoL, n (%)	P value
Sex			
Feminine	260 (31.9)	228 (28.0)	0.0309*
Masculine	149 (18.3)	178 (21.8)	
Age range (years)			
11-13	255 (31.3)	238 (29.2)	0.2766
14-16	154 (18.9)	168 (20.6)	
Skin color			
White	158 (19.6)	175 (21.7)	0.4064
Brown	199 (24.7)	186 (21.7)	
Black	52 (6.6)	45 (5.7)	
Previous orthodontic treatment			
Yes	82 (10.1)	108 (13.2)	0.0270*
No	327 (40.1)	298 (36.6)	
Desire to correct teeth			
Yes	390 (47.8)	355 (43.6)	<.0001*
No	19 (2.3)	51 (6.3)	
Dental Aesthetic Index (DAI)			
≤ 25 (no treatment necessary)	246 (30.18)	246 (30.18)	0.9539
>25 (treatment necessary)	163 (20.0)	161 (19.75)	
Victim of bullying			
Yes	246 (30.2)	147 (18.0)	<.0001*
No	163 (20.0)	259 (31.8)	
Frequency of suffering bullying			
One time	73 (18.6)	64 (16.3)	0.0170*
Several times	103 (26.2)	58 (14.8)	
Almost everyday	42 (10.7)	15 (3.8)	
Several times a day	28 (7.1)	10 (2.5)	
Consequences of bullying			
No consequences	110 (28.0)	104 (26.5)	<.0001*
Some bad consequences	106 (27.0)	34 (8.6)	
Terrible consequences	18 (4.6)	4 (1.0)	
Had to change school	12 (3.0)	5 (1.3)	
Type of bullying suffered			
Emotional	17 (4.4)	19 (4.9)	0.1289
Verbal	110 (28.0)	73 (18.5)	
Physical	33 (8.4)	22 (5.6)	
Racist	35 (9.0)	13 (3.3)	
Sexual	7 (2.0)	4 (1.0)	
More than one response	43 (10.6)	15 (3.9)	
No response	1 (0.2)	1 (0.2)	
Victim was already antagonist of bullying			
Yes	75 (19.1)	52 (13.2)	0.3162
No	171 (43.5)	95 (24.2)	

*statistically significant.

After the association analysis, the variables sex, skin color, desire to fix teeth, previous orthodontic treatment, frequency and consequences of bullying were assessed in the logistic regression model. Multivariate analysis considering the outcome OHRQoL showed that adolescents who reported consequences resulting from episodes of bullying had three times greater chance of having a bad OHRQoL. Another important fact was the protective effect of previous orthodontic treatment, since adolescents who had undergone treatment were two times less likely to have a bad OHRQoL (Table 5).

**Table 5:** Logistic regression analysis considering the poor “oral health-related quality of life” outcome with the dependent variables.

VARIABLES		OR	CI 95%
Sex	Feminine	1.172	0.745 - 1.845
	Masculine	1	
Skin color	Non-white	1.331	0.856 - 2.069
	White	1	
Desire to correct teeth	Yes	1.226	0.524 - 2.865
	No	1	
Frequency of suffering bullying	One time	1.482	0.935 - 2.340
	More than once	1	
Consequences of bullying	With consequences	3.042	1.715 - 4.831*
	Without consequences	1	
Previous orthodontic treatment	No	2.191	1.302 - 3.687*
	Yes	1	

* statistically significant.

## DISCUSSION

The results of this research are of great importance, since no previous study examined the influence of bullying on the OHRQOL of Brazilian adolescents. Students who reported negative consequences of episodes of bullying were three times more likely to have a bad OHRQoL. Only one study in Jordan^20^ and another one in the United Kingdom^24^ showed this negative impact of bullying on the OHRQoL. In the first study, the authors found this association in 11- and 12-year-old students who self-reported episodes of bullying motivated by dentofacial characteristics; however, no oral examination for the presence of malocclusion was performed^21^. On the other hand, in the study conducted in the UK, the population consisted of adolescents who sought orthodontic treatment^21^. Thus, we can highlight the influence of bullying on OHRQoL, regardless of whether the sample was taken from the general population or a population in need for orthodontic treatment.

Assessments of OHRQoL are important for subjective evaluations of the population,^4^ as these assessments involve a multidimensional perception of the individual, and the findings of these assessments should be taken into consideration because concerns such as dental aesthetics and psychological well-being are often presented as reasons for seeking orthodontic treatment during childhood and adolescence.^20^

Bullying can have devastating consequences on the emotional state of adolescents. Low self-esteem, impaired school performance, depression, loneliness, insecurity, and shyness are commonly reported in the literature as sequelae of such situations.^22^ ^,^ ^23^ In this context, interventions to prevent bullying are necessary, because episodes of bullying in adolescence can have life-long emotional consequences. In more serious cases of persecution, depression can severely compromise the emotional state of the victim and, in a desperate attempt to stop the persecution, the victim may even attempt suicide.^24^

A notable finding in this study is that the factors subsequent to bullying had a greater influence on the OHRQoL than the actual event itself, because the need for orthodontic treatment had no statistically significant association with OHRQoL. This result differs from most studies that evaluated this condition and found an association between malocclusion and bad OHRQoL.^21^ ^,^ ^25^ In contrast, almost all the adolescents studied reported the desire to fix the teeth to improve their appearance, regardless of the need for treatment. This variable had a significant association with oral health-related quality of life, suggesting the adolescents’ great concern with body image. 

As previously reported in other studies,^26^ there was a positive impact of previous orthodontic treatment on OHRQoL, and those who had not previously been treated were twice as likely to have a bad OHRQoL. This fact is interesting because the study was conducted in the general population, at school, and not with patients seeking orthodontic treatment, which could be a bias. Surprisingly, the present study showed no association between need for orthodontic treatment and victims of bullying. There is a complex relationship between the presence of malocclusion, psychological harassment, and OHRQoL. The cause-effect relationship in abused individuals is still unclear, and a combination of factors act synergistically to cause a negative impact on the psychological well-being of the individual.^14^ ^,^ ^27^ It is assumed that the lack of association between these variables can be justified by the fact that this research was conducted outside the clinical setting. The high prevalence of bullying in this study encompasses different types of students with great physical and emotional diversity, and does not show a common pattern among the teens. Adolescence is a period of great changes^10^ ^,^ ^11^, and the concepts established in this age have repercussions on behavioral relationships in adulthood and can have negative consequences both in the professional field and in the affective zone.^28^ ^-^ ^30^

Some limiting factors should be considered, such as the fact that the results of this study cannot be generalized to other locations; however, it is important to emphasize the need to compare the results of this study with those of other studies with similar goals conducted in Brazil and other countries. It should also be considered the low adhesion of the target population. Although the sample was representative, it was obtained a low rate of returns of parents/guardians’ authorizations. We cannot state whether the authorizations were given to those responsible for the students; according to a report of the coordination of participating schools, the age range of the group studied does not show great interest in extra-curricular subjects. Moreover, unlike other countries, culturally, Brazilians do not have the habit of participating in scientific research; the government does not encourage this practice, which is important for both the scientific environment and the community. However, it is of great importance for the population to highlight and measure the problem related to collaboration in directing targeted strategies for preventive action. Both the school and the family environment play an important role in supporting these adolescents.

## CONCLUSION

There was an association between the OHRQoL and bullying variables. Adolescents who reported negative consequences after bullying episodes were three times more likely to have bad OHRQoL. There was no statistically significant association between the need for orthodontic treatment and OHRQoL, but adolescents who have a history of orthodontic treatment prior to the research showed a protective effect against bad OHRQoL.
